# Hepcidin peptide controls the inflammatory response induced by betanodavirus infection and improves European sea bass (*Dicentrachus labrax*) survival

**DOI:** 10.1007/s42995-024-00262-w

**Published:** 2025-01-23

**Authors:** Laura Cervera, Marta Arizcun, Luis Mercado, Alberto Cuesta, Elena Chaves-Pozo

**Affiliations:** 1 Immunobiology for Aquaculture Group, Department of Cell Biology and Histology, Faculty of Biology, University of Murcia, 30100 Murcia, Spain; 2 Centro Oceanográfico de Murcia (COMU-IEO), CSIC, Carretera de la Azohía s/n, Puerto de Mazarrón, 30860 Murcia, Spain; 3 Grupo de Marcadores Inmunológicos, Laboratorio de Genética E Inmunología Molecular, Instituto de Biología, Pontificia Universidad Católica de Valparaíso, 2373223 Valparaíso, Chile

**Keywords:** Antimicrobial peptides (AMPs), Hepcidin, Betanodavirus, Immunity, European sea bass, Aquaculture

## Abstract

Nervous necrosis virus (NNV) is the etiological agent of viral encephalopathy and retinopathy in many fish species, including European sea bass (*Dicentrachus labrax*) and is of great economic losses to fish farmers. To solve this problem in fish production, antimicrobial peptides (AMPs) have been identified as potential candidates for NNV treatment in aquaculture. Hepcidin (Hamp) is one of the most promising AMPs. Thus, we aimed to evaluate the therapeutic application of Hamp synthetic peptide after NNV challenge. Hamp was able to significantly increase survival rates and ameliorate clinical signs of the disease, though the viral levels, determined by viral replication and immunolocalization, were not affected. Synthetic Hamp increased the immunoglobulin M (IgM) and AMP protein levels in serum and some tissues respect to the levels found in NNV-infected fish. However, Hamp peptide decreased the NNV-induced bactericidal activity. At the gene level, Hamp exerted anti-inflammatory properties, reducing the pro-inflammatory response orchestrated by NNV, probably preventing neuronal damage. Apart from this, Hamp up-regulated the expression of adhesion molecules that facilitated the recruitment of immune cells, namely T helper and B cells, probably to orchestrate the adaptive response. To conclude, Hamp immunomodulatory properties and therapeutic application against NNV are very promising for its use in aquaculture.

**Supplementary Information:**

The online version contains supplementary material available at 10.1007/s42995-024-00262-w.

## Introduction

The crucial role of aquaculture as a sustainable way of producing human food and reducing the pressure exerted on marine resources has made this sector one of the most prosperous worldwide (FAO 2020). Nevertheless, the intensive culture conditions of fish favors disease outbreaks, including those of viral origin (Kibenge 2019). Nervous necrosis virus (NNV) is the agent causing the viral encephalopathy and retinopathy disease in more than 180 species of teleost fish, demonstrating a low host specificity (Low et al. 2017). Several species of great interest for the aquaculture industry are very susceptible to this infection, and include European sea bass (*Dicentrarchus labrax*), Asian sea bass (*Lates calcarifer*), orange-spotted grouper (*Epinephelus coioides*) and Atlantic cod (*Gadus morhua*). NNV causes high mortalities in larvae and juvenile stages, while adults and breeders usually survive and act as reservoirs to spread the virus. NNV is a non-enveloped small virus composed of two single-stranded RNA molecules: RNA1, which codifies for the RNA-dependent RNA-polymerase (RdRp; also named protein A), and RNA2, which encodes the capsid protein (Cp) (Low et al. 2017). During NNV infection, a subgenomic RNA molecule called RNA3 is also produced, which codifies for the non-structural proteins B1 and B2, which are involved in viral pathogenesis (Low et al. 2017). Effective diagnostic methods and the molecular mechanisms involved during infection have been extensively researched (Bandín and Souto 2020). Unfortunately, very few studies have evaluated potential treatments (Costa and Tho^2020^mpson 2016). Among these studies, antimicrobial peptides (AMPs) have been postulated as potential treatments of NNV (Chia et al. 2010; León et al. 2020).

AMPs are a heterogenous and diverse group of proteins characterized by their short amino acidic sequence, amphipathic and cationic properties and predominant alpha helix secondary structures (Katzenback 2015). Interestingly, AMPs show little cytotoxicity and low potential for inducing pathogen resistance (Cabello et al. 2016). These properties suggest that AMPs are excellent candidates for use in aquaculture without adversely impacting the marine environment. Here, we focused on one of the most characterized AMPs in teleost fish, hepcidin (Hamp). Hamp has dual roles in animals being a key molecule in iron absorption as well as its properties as AMP. The direct antimicrobial roles of Hamp against viruses, bacteria and parasites and in modulating immune responses have been well detailed in several marine fish species, e.g., sea bass or tilapia (Álvarez et al. 2016, 2022; Cervera et al. 2023; Wang et al. 2010a). Hepcidin appears to exert a major role in the regulation of the fish inflammatory response, though studies in this regard are controversial. For example, in zebrafish (*Danio rerio*), the administration of Hamp resulted in the up-regulation of the anti-inflammatory interleukin (*il*)*10*, whereas in European sea bass the pro-inflammatory cytokines *il6*, *il1b* and tumor necrosis factor alpha (*tnfa*) were up-regulated in both in vivo and in vitro experiments (Álvarez et al. 2022; Cervera et al. 2023; Neves et al. 2015). In addition, Hamp administration regulates the expression of several chemokines or interleukins related to cell recruitment and infiltration (Hsieh et al. 2010; Pan et al. 2011b) as well as the production of other AMPs (Cervera et al. 2023; Pan et al. 2011a). Interestingly, although AMPs are considered major arms of the innate immune response, they also bridge innate and adaptive immunity in mammals (van der Does et al. 2019). This has also been proposed in European sea bass and grouper (*Epinephelus lanceolatus*) (Cervera et al. 2023; Ting et al. 2019).

Focusing on NNV infection, our group has described that in European sea bass, the infection triggered an increased Hamp gene expression and protein production (Valero et al. 2015, 2020). Moreover, the administration of Hamp synthetic peptide at or before the time of infection had a positive impact on the survival of medaka (*Oryzias latipes*) (Wang et al. 2010a) and European sea bass (Cervera et al. 2024), although the administration of Hamp-expressing plasmid did the opposite in European sea bass (Cervera et al. 2023). Taking into consideration all the information available about preventaive Hamp applications in European sea bass against NNV infections, we further evaluated its potential therapeutic application in aquaculture. Thus, Hamp synthetic peptide was administered to European sea bass after NNV infection to evaluate disease protection and the development of immunity. Data will shed light in the Hamp anti-NNV applications as well as its ability to modulate host immune responses.

## Materials and methods

### Animals

Apparently, healthy juvenile European sea bass (*Dicentrarchus labrax* L) with a mean body weight of 5.94 ± 0.27 g were bred at COMU-IEO, CSIC facilities using standard culture protocols as described previously (Cervera et al. 2024). The physiochemical parameters were: 38‰ salinity, temperature of 25 ± 1 °C, photoperiod of 12:12 light:dark and ad libitum diet supply. Ethical management protocols and authorization by local authorities were obtained for all the experiments (Permit Numbers A13210701 and A13202602).

### Hepcidin peptide and NNV production

Mature European sea bass hepcidin (Hamp 2, variant 1; accession number KJ890397.1; HSSPGGCRFCCNCCPNMSGCGVCCTF) was synthesized by GeneScript (Purity ≥ 90%), dissolved in pure water at 1 mg/mL and the aliquots frozen. The bioactivity of the selected Hamp peptide has already demonstrated antibacterial (Neves et al. 2015) and immunomodulatory properties (Cervera et al. 2024).

NNV (strain It/411/96; genotype RGNNV) was propagated in the E-11 cell line as described elsewhere (Iwamoto et al. 2001). Briefly, the E-11 cells were cultured in 25 cm^2^ tissue culture flasks (Nunc) at 25 °C using Leibovitz’s L-15 medium (Gibco) supplemented with 10% fetal bovine serum (FBS). E-11 monolayers were infected with NNV at a multiplicity of infection (MOI) of 1 with culture medium containing 2% (v/v) FBS until the cytopathic effect (CPE) was extensive. Then, NNV stocks were titrated using E-11 cells cultured in 96-well microtiter plates (Nunc) in triplicate. The viral dilution infecting 50% of the cell cultures (TCID_50_) was calculated after Reed and Müench (1938).

### Experimental design

To evaluate the therapeutic applications of Hamp against NNV infection, fish were randomly divided into three experimental groups (55 fish/group): controls, infected with NNV, and infected with NNV and then treated with Hamp (NNV + Hamp). For this, fish from NNV and NNV + Hamp groups were injected intramuscularly (im) with 50 µL of NNV (TCID_50_/mL = 2.8 × 10^6^), whereas the control group was injected with phosphate buffer (PBS). After 24 h of infection, fish from the NNV + Hamp group were im injected with 50 µL at ∼ 1 µg Hamp per gram of fish. Fish from the other groups (controls and NNV) were injected with 50 µL of PBS. Previous to being handled, all fish were captured and anesthetized with 40 µL/L of clove oil in marine water. Injection was performed with an insulin syringe approximately in the same area of the dorsal muscle, following the same handling conditions.

To analyze the mortalities and clinical signs of infection, a ranked severity signal from 1 (low welfare impact) to 4 (extremely severe signs) were obtained as described previously (Cervera et al. 2024) with daily recordings. Three consecutive days with no mortalities in each tank determined the end of the experiment.

### Sampling

Fish were sampled (*n* = 6/group) three days after NNV infection (dpi) and two days after Hamp treatment (dpt). Briefly, anesthetized specimens, as described above, were completely exsanguinated and rapidly decapitated. Serum samples were obtained after centrifugation of blood obtained from the caudal vein (10,000 *g* for 10 min at 4 °C) and stored at − 80 °C. Fragments from muscle, spleen, brain and head kidney (HK) tissues were immediately frozen and stored at − 80 °C. For histological analyses, brain samples were collected (*n* = 3/group) and processed for light microscopy.

### NNV detection by immunohistochemistry (IHC)

Brain samples were fixed in Bouin’s solution (16 h at 4 °C), dehydrated and embedded in paraffin (Paraplast Plus; Sherwood Medical) using standard protocols previously described (Chaves-Pozo et al. 2005). Brain sections of 5 μm thickness were subjected to an indirect IHC method using a commercial anti-RGNNV antiserum (Abcam: 26812), which specifically binds to the capsid protein of NNV, using a previously described protocol with slight modifications (Chaves-Pozo et al. 2021). Slides were examined with an Eclipse E600 light microscope (Nikon). Images were obtained with an Olympus SC30 digital camera (Olympus soft imaging solutions). For each fish (*n* = 3 fish/group), three different sections and four optical areas/section were used to determine the area of anti-NNV immunostaining. Images at ×20 magnification were processed with ImageJ software.

### ELISA analysis

Brain, spleen and HK samples were homogenized in PBS (1 g of tissue/mL of PBS) and centrifuged; the resultant supernatant was used later. Total proteins present in serum, brain, spleen and HK homogenates were measured using the Bradford assay.

The indirect ELISA technique was used to detect the following proteins: NNV capsid protein in brain homogenates; total immunoglobulin M (IgM) in serum and brain homogenates; and AMPs (Nkl, Hamp and Dic) in serum, brain, HK and spleen homogenates. To perform the ELISAs, 100 µL/well of coating buffer (CB; 100 mmol/L bicarbonate/carbonate, pH 9.6) containing 40 μg of total proteins from tissue homogenates or 1:1000 dilution of sera were placed in 96 Maxisorp flat-bottomed microtiter plates (Nunc) and incubated overnight at 4 °C. After three washes with PBS containing 0.2% Tween-20 (PBS-T), 3% bovine serum albumin (BSA) in PBS was used to block the unspecific binding with 2 h incubation. Afterward, samples were incubated with their respective primary antibody: polyclonal anti-RGNNV (Abcam: 26,812) for NNV detection; polyclonal anti-Nkl, anti-Hamp or anti-Dic antiserum for AMPs detection or anti-European sea bass IgM (Aquatic Diagnostics) at their optimal dilutions of 1:5,000, 1:200, 1:200, 1:200 and 1:100, respectively (Valero et al. 2020). After washing with PBS-T, samples were incubated with the respective anti-mouse or anti-rabbit IgG-HRP (Thermo Fisher Scientific) for 1 h. The reaction was developed using 3,3′,5,5′-tetramethylbenzidine (TMB; Sigma-Aldrich) and stopped with 2 mol/L sulfuric acid. An absorbance reader (Multiskan GO, ThermoFisher Scientific) was used at 450 nm. Positive and negative (without serum or primary antisera) controls were always included in the reactions. Absorbance was normalized to the µg of proteins used.

### Antibacterial activity

The antibacterial activity of serum, and HK, brain and spleen homogenates was determined by evaluating their effects on the bacterial growth of *Vibrio harveyi* (Vh; strain Lg 16/100) as previously described (Cervera et al. 2023). Aliquots (10 μL) of sample from serum or tissue homogenates (1 mg/mL of protein) were used. The sample and bacteria were replaced by Tryptic Soy Broth (TSB) in negative control wells (0% bactericidal activity, 0% bacterial growth), while replacing only the sample used as a positive control (0% bactericidal activity, 100% bacterial growth).

### Gene expression analysis

One μg of total RNA was isolated using TRIzol reagent (Invitrogen) and processed to synthetize the first-strand cDNA free of genomic contamination as previously described (Cervera et al. 2023). qPCR was performed in individual cDNA samples with a Quant Studio 5 instrument (Applied Biosystems) using SYBR Green PCR Core Reagents (Applied Biosystems) as described by Cervera et al. (2023). For each mRNA, gene expression was corrected by the geometric mean of three housekeeping genes (elongation factor 1 alpha (*ef1a*), ribosomal protein 18 (*rps18*) and ribosomal protein L13 alpha (*l13a*)) and expressed as 2^−Δ*Ct*^, where ΔCt is determined by subtracting the housekeeping genes Ct values from the target Ct in each sample (Pfaffl 2001). The genes analyzed were grouped in the following categories: AMPs, inflammatory-related cytokines, cell recruitment, leucocyte-type markers, antiviral response and NNV markers. Gene names and primer sequences are included in Supplementary Table S1. Negative controls with no template were always included in the reactions.

### Statistical analysis

The Kaplan–Meier method was used to represent survival and the log-ranked (Mantel–Cox) test to determine statistical differences. All data are presented as mean ± standard error of the mean (SEM). Shapiro–Wilk and Levene tests were used to validate the assumed parametric conditions of the data and in this case ANOVA or Student’s *t* tests were used. When parametric assumptions were not demonstrated a *U* Mann–Whitney or a Kruskal–Wallis test was applied. SPSS 24 and GraphPad Prism 8 software were used, and the minimum level of significance fixed at 0.05 (*P* ≤ 0.05).

## Results

### Hamp increases the European sea bass survival upon NNV infection

**Fig. 1:**
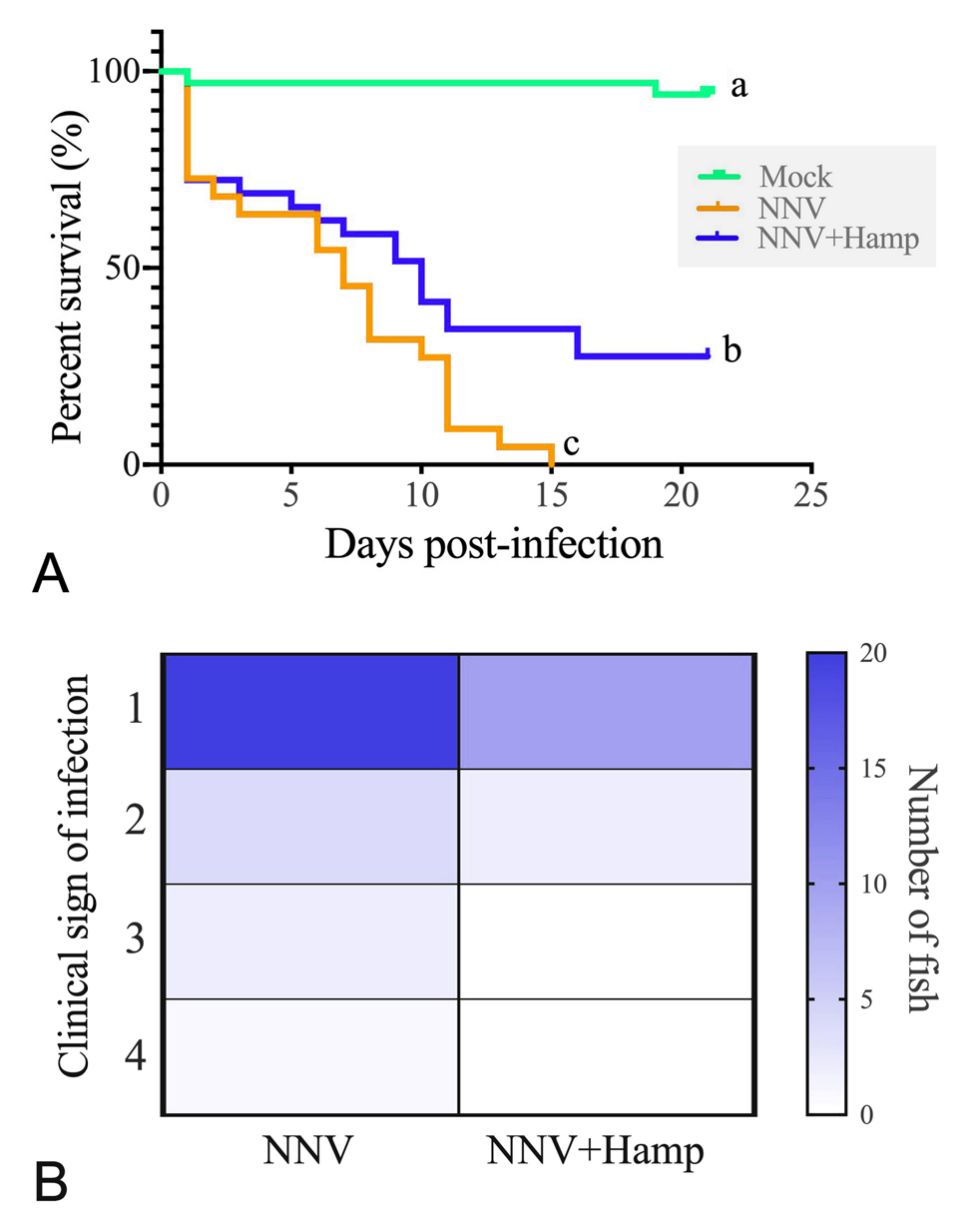
Therapeutic administration of synthetic hepcidin (Hamp) peptide improves the survival of European sea bass against nervous necrosis virus (NNV). European sea bass juveniles were infected intramuscularly with NNV (TCID_50_/fish = 2.8 × 10^6^) and one day later injected with PBS or Hamp (∼ 1 μg peptide per g of fish). A control group was injected twice with only PBS. **A** Kaplan–Meier survival curves showing the proportion of European sea bass survivors upon NNV infection. Different letters indicate differences between groups according to a log-rank test (*P* ≤ 0.05). **B** Heat map representing the cumulated number of fish showing clinical signs of NNV disease according to their severity: (1) changes in the color of the skin, slower rhythm of swimming and/or slower reaction to external stimuli such as feeding; (2) alterations in the swimming balance and/or erratic swimming spasms; (3) continuous erratic swimming; and (4) complete incapacity to keep balance, swim and/or move without external stimuli

**Fig. 2:**
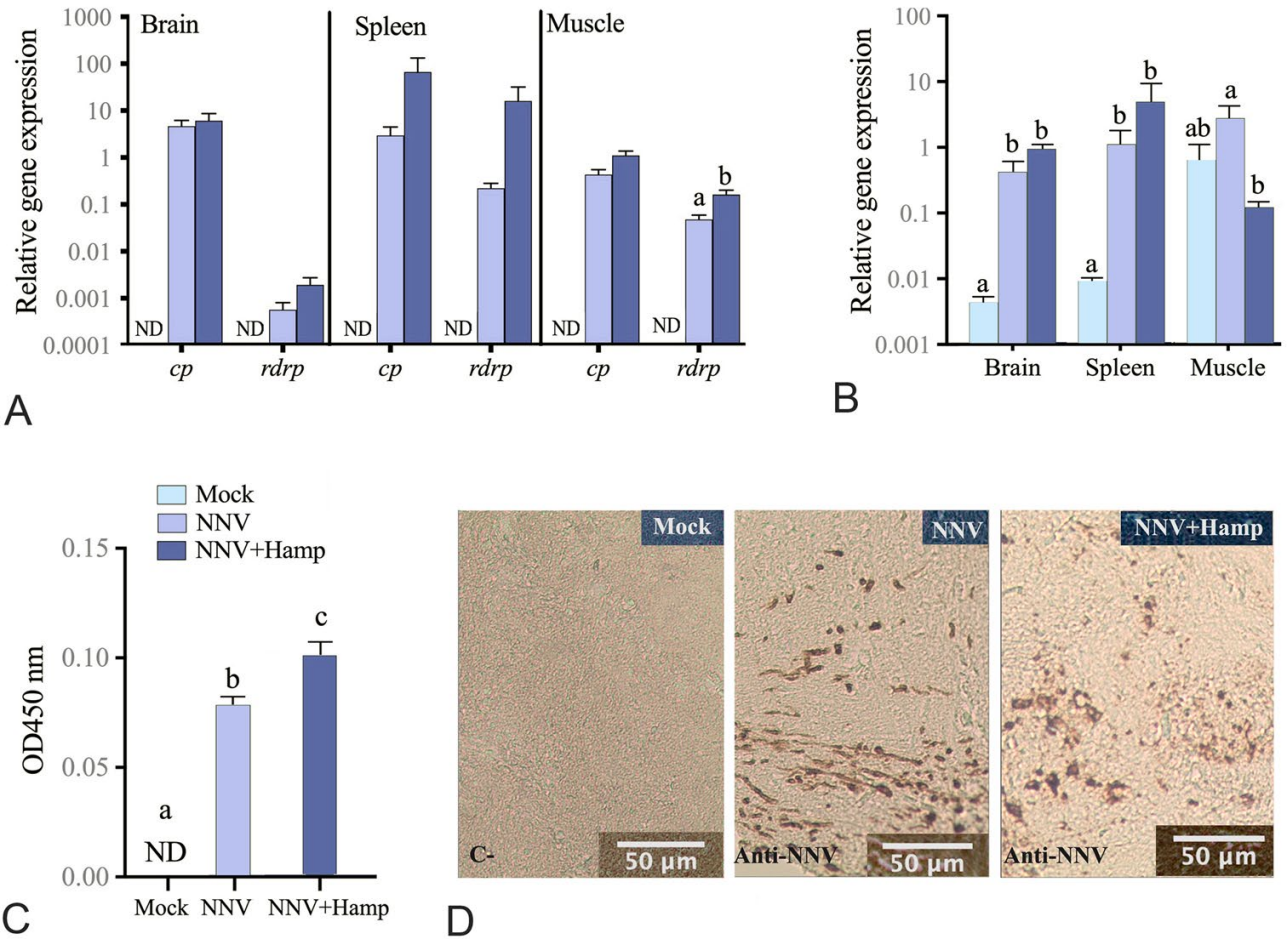
Brain NNV-Cp levels are increased by Hamp. European sea bass juveniles were infected intramuscularly with nervous necrosis virus (NNV; TCID_50_/fish = 2.8 × 10^6^) and one day later treated with Hamp (∼ 1 μg peptide per g of fish; Hamp + NNV group) or vehicle (PBS; NNV group). A control group was injected twice with only PBS. Brain, spleen and muscle samples (*n* = 6) were obtained two days after Hamp treatment. **A** Transcription levels of NNV *rdrp* and *cp* genes. **B** Transcription levels of the antiviral marker *mx*. **A**, **B** Data represent the mean relative gene expression ± SEM (*n* = 6) obtained by real-time PCR. **C** Brain levels of NNV capsid protein (Cp) determined by ELISA. Data represent the mean optical density (OD) at 450 nm ± SEM (*n* = 6). **A**–**C** Different letters indicate significant statistical differences among the experimental groups according to ANOVA, followed by Tukey’s post hoc or Student’s *T* tests (*P* ≤ 0.05). ND, no detection. **D** Sections of brain immunostained with the anti-RGNNV serum. Brown cells correspond to NNV-infected cells

Hamp administration significantly increased (*P* = 0.0184) the survival of sea bass infected with NNV compared to the controls (Fig. 1A). Moreover, the severity of the clinical signs was also decreased (Fig. 1B). However, similar transcription levels of viral genes (Fig. 2A) and the antiviral *mx* (Fig. 2B) were observed in the brain and spleen of fish from the NNV and NNV + Hamp groups. Exceptionally, the transcription levels of NNV *rdrp* were higher, whereas those of *mx* were lower, in the muscle of Hamp-treated fish infected with NNV (Fig. 2A, B). In contrast, analysis of the viral Cp protein by ELISA revealed significantly higher levels in the brain of the NNV + Hamp group than in the NNV group (Fig. 2C). Despite that, the percentage of the total area stained with anti-NNV in brain was similar in both groups (NNV group = 5.8 ± 1.3%, and NNV + Hamp group = 4.8 ± 1.3%; Fig. 2D).

### Hamp alters the immune response triggered by NNV infection

**Fig. 3:**
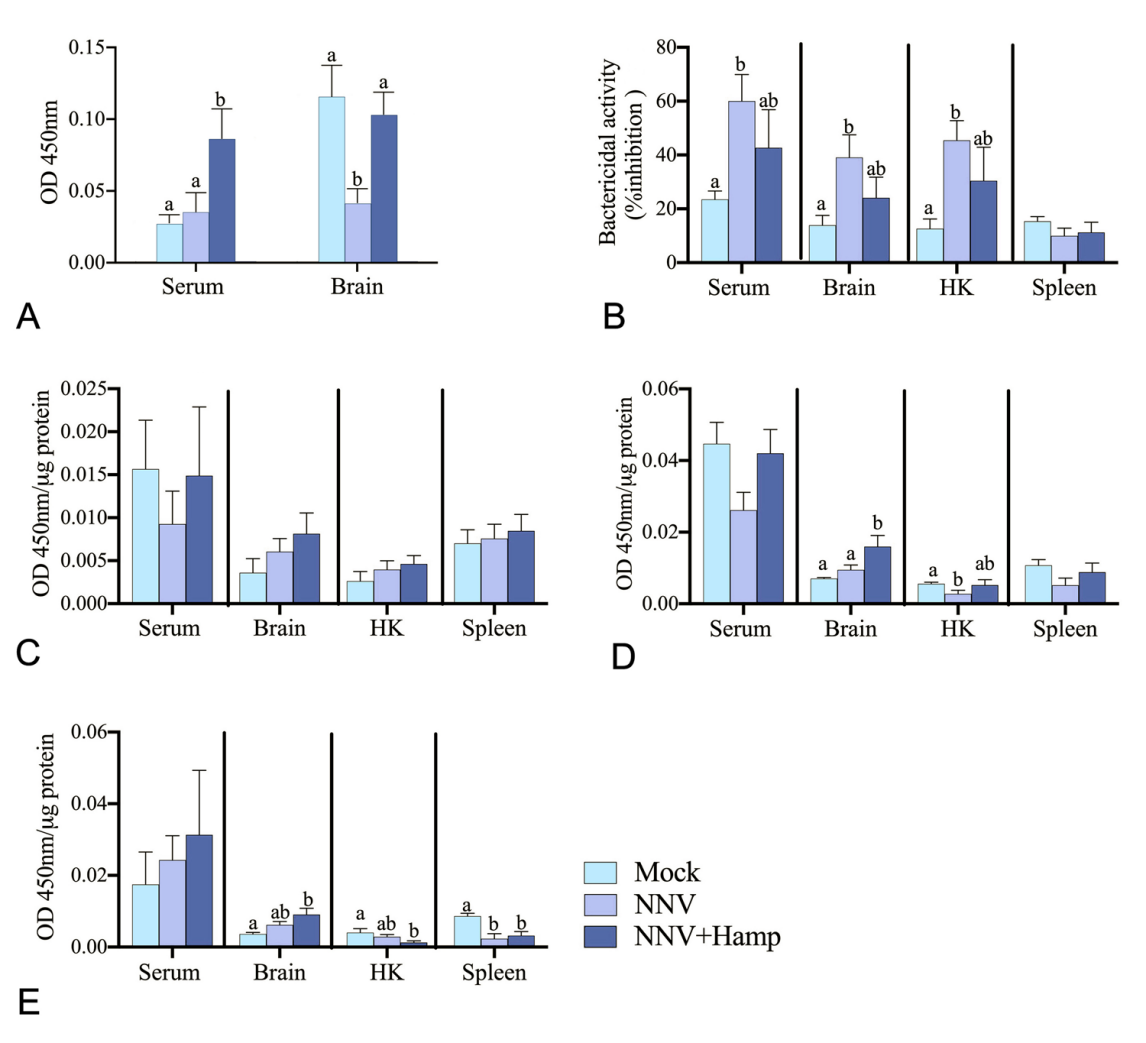
Immune parameters are slightly modulated by Hamp treatment upon NNV challenge. European sea bass juveniles were infected intramuscularly with nervous necrosis virus (NNV; TCID_50_/fish = 2.8 × 10^6^) and one day later treated with Hamp (∼ 1 μg peptide per g of fish; Hamp + NNV group) or vehicle (PBS; NNV group). A control group was injected twice with only PBS. Serum, brain, head kidney (HK) and spleen samples (*n* = 6) were obtained two days after Hamp treatment. **A** Total IgM levels in serum and brain. **B** Antibacterial activity against *Vibrio harveyi*. (**C-E**). Protein levels of Hamp (**C**), Nkl (**D**) and Dic (**E**). In all cases, data represent the mean ± SEM (*n* = 6), while different letters indicate significant statistical differences among groups according to ANOVA, followed by Tukey’s post hoc test (*P* ≤ 0.05). Absorbance was normalized to the µg of proteins used

We evaluated some parameters of the humoral immune response, including IgM levels, bactericidal activity and AMP levels. Serum total IgM levels were significantly increased in Hamp-treated specimens in comparison to the control and NNV groups (Fig. 3A). However, in the brain, the levels of total IgM in the NNV group were significantly reduced compared to the controls, but treatment with Hamp restored them to control levels (Fig. 3A). Regarding the antibacterial activity, NNV infection resulted in a significantly increased bactericidal activity in European sea bass serum, brain and HK homogenates when compared to the controls, but therapy with synthetic Hamp reduced it to non-significant levels (Fig. 3B). To ascertain whether AMPs are involved in this antimicrobial activity, we determined the levels of Nkl, Hamp and Dic AMPs by indirect ELISA. Surprisingly, Hamp levels were not affected to a significant extent in any of the groups and tissues analyzed (Fig. 3C). Regarding NKl, treatment with Hamp significantly increased the levels in the brain with respect to the fish infected alone, whereas the NNV-reduced levels were restored in the HK (Fig. 3D). In addition, the levels of Dic in the NNV + Hamp group were significantly increased in brain and decreased in HK and spleen when compared with the controls (Fig. 3E). No significant differences were observed between the NNV and Hamp + NNV groups.

### Synthetic Hamp up-regulates several immune-related genes in the brain

**Fig. 4:**
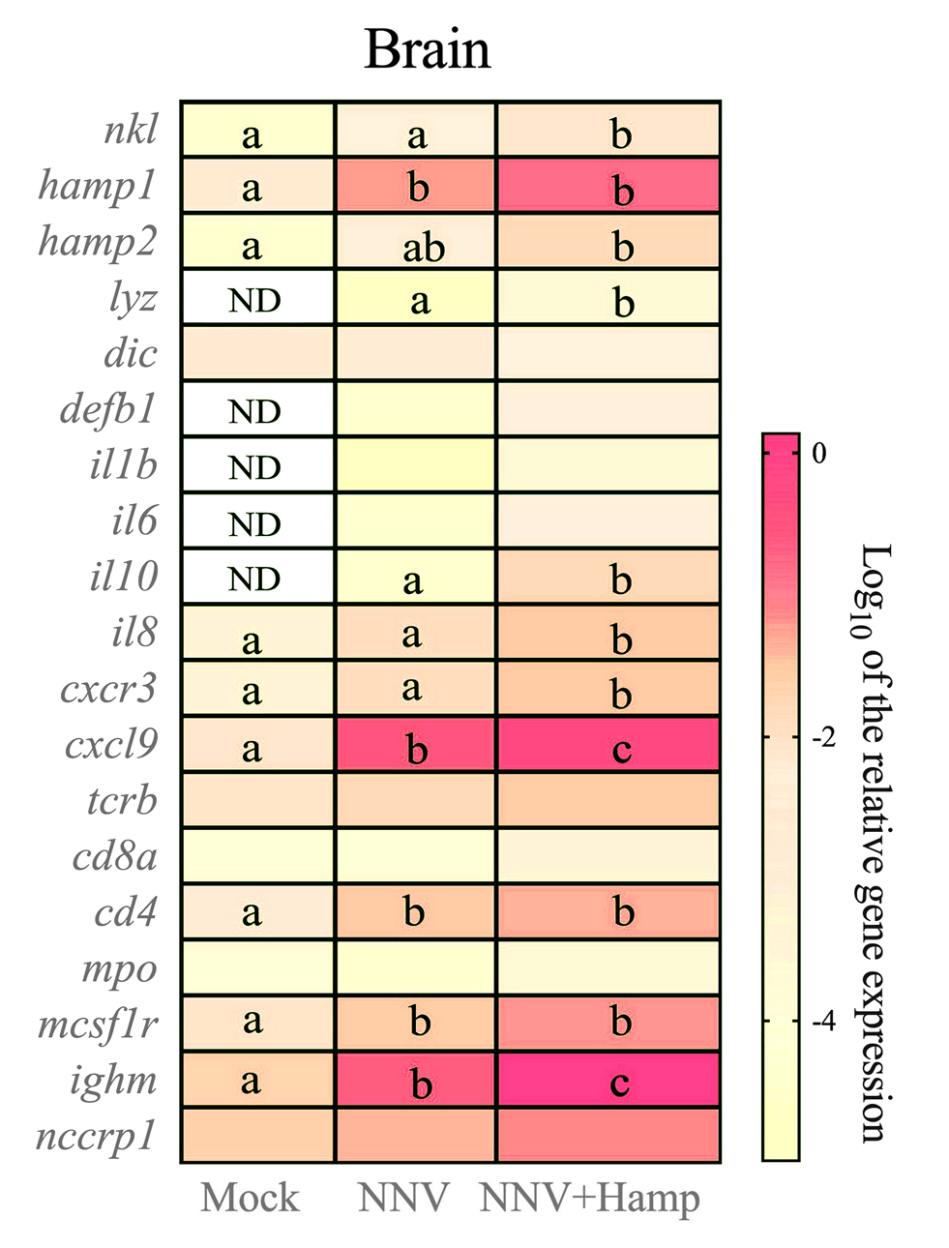
Hamp treatment after NNV challenge in European sea bass stimulates several immune-related genes in the brain. European sea bass juveniles were infected intramuscularly with nervous necrosis virus (NNV; TCID_50_/fish = 2.8 × 10^6^) and one day later treated with Hamp (∼ 1 μg peptide per g of fish; Hamp + NNV group) or vehicle (PBS; NNV group). A control group was injected twice with only PBS. Brain, muscle, head kidney (HK) and spleen samples (*n* = 6) were obtained two days after Hamp treatment. Data represent the mean relative gene expression ± SEM (*n* = 6). Different letters indicate significant statistical differences among groups according to ANOVA, followed by Tukey’s post hoc test (*P* ≤ 0.05). Gene abbreviations are described in Supplementary Table S1

We evaluated the transcription of several immune-related genes in the NNV replication (brain), the injection site (muscle) and immune (HK and spleen) tissues. In brain, NNV infection resulted in significant transcriptional induction of AMP *hamp1*, chemokine *cxcl9* and cell markers for T helper (*cd4*), B cells (*ighm*) and macrophages (*mcsf1r*) compared to controls though the expression of the AMPs *lyz* and *defb1* and the pro-inflammatory cytokines *il1b*, *il6* and *il10* shifted from not detected to detectable (Fig. 4). Compared to NNV alone, Hamp treatment significantly up-regulated the expression of AMPs *nkl* and *lyz,* anti-inflammatory interleukin *il10,* chemokines *il8*, *cxcr3* and *cxcl9* and B cell marker *ighm* genes, whereas other genes remained at similar levels to the NNV infected group (Fig. 4).

**Fig. 5:**
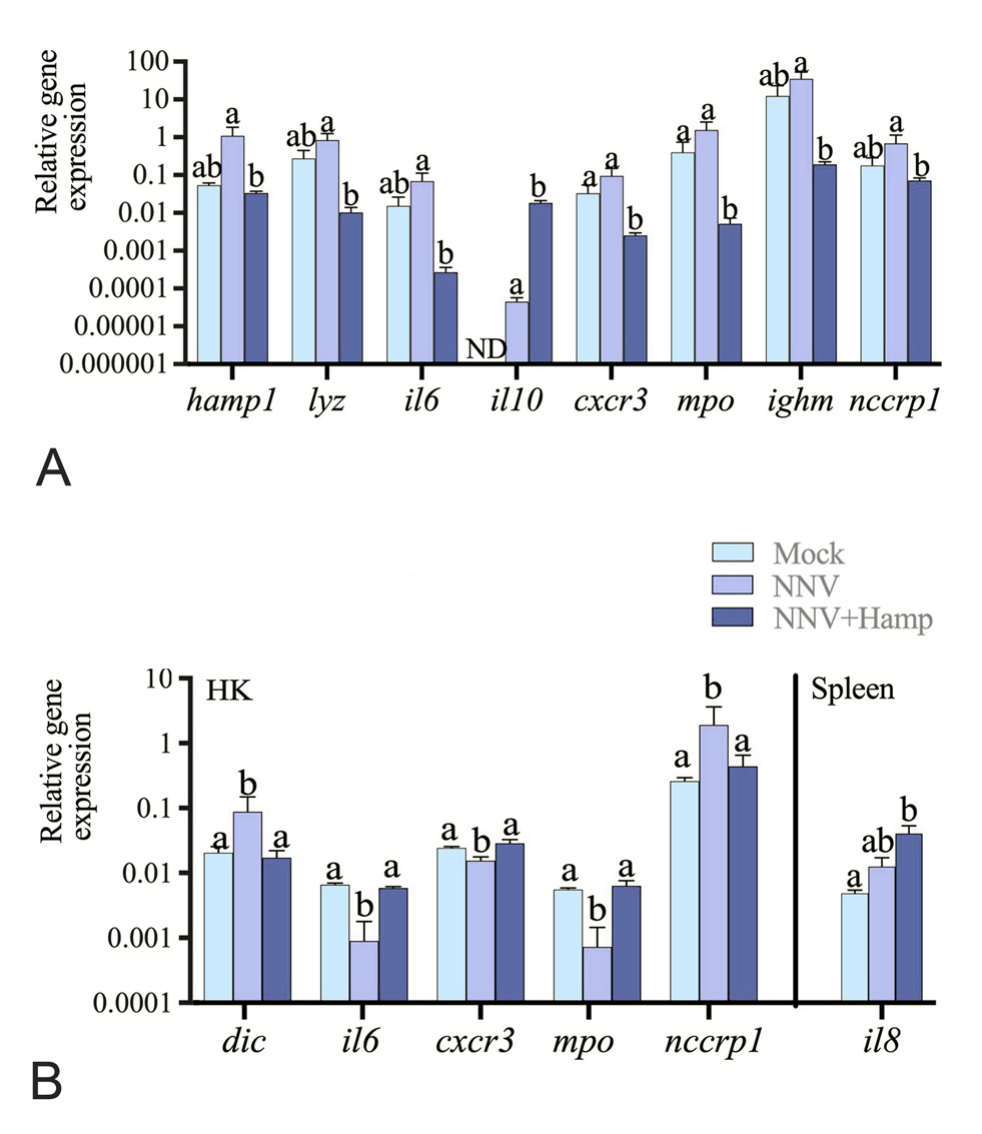
Hamp treatment after NNV challenge in European sea bass stimulates several immune-related genes in muscle and immune organs. European sea bass juveniles were infected intramuscularly with nervous necrosis virus (NNV; TCID_50_/fish = 2.8 × 10^6^) and one day later treated with Hamp (∼ 1 μg peptide per g of fish; Hamp + NNV group) or vehicle (PBS; NNV group). A control group was injected twice with only PBS. Brain, muscle, head kidney (HK) and spleen samples (*n* = 6) were obtained two days after Hamp treatment. **A**, **B**. The relative gene expression levels of immune-related genes in muscle (**A**) and immune organs, head kidney (HK) and spleen (**B**). In all cases, data represent the mean relative gene expression ± SEM (*n* = 6). Different letters indicate significant statistical differences among groups according to ANOVA, followed by Tukey’s post hoc test (*P* ≤ 0.05). Gene abbreviations are described in Supplementary Table S1

At the injection site, we observed a generalized down-regulation in the transcription of immune markers in the NNV + Hamp group compared to the NNV-infected group (*hamp1*, *lyz*, *il6*, *cxcr3*, *mpo*, *ighm* and *nccrp1* genes), whereas only *il10* was up-regulated (Fig. 5A). In immune tissues, treatment with Hamp led to reversion of the transcriptional profile of all the NNV-regulated genes (*dic*, *il6*, *cxcr3*, *mpo* and *nccrp1*), whereas a large up-regulation in the spleen *il8* gene expression was recorded when compared to the controls (Fig. 5B). Genes without significant regulation in muscle, HK or spleen have not been shown.

## Discussion

Viral infections have devastating consequences in fish farms, such as diminished fish quality and production, leading to severe economic losses. Besides, the use of therapeutic antiviral agents for fish use is currently restricted to laboratory trials, though their implementation in practical aquaculture could be extremely interesting (Ahmed et al. 2019). Regarding NNV and its importance in the Mediterranean area, increasing studies are trying to solve this problem by developing new therapeutic compounds (Chia et al. 2010; León et al. 2020; Morick and Saragovi 2017; Sushila et al. 2018). To fulfill this goal, AMPs have occurred as among the most promising agents due to their dual role as lytic molecules and immunomodulators as well as the lack of cytotoxicity or development of bacterial resistance (Shabir et al. 2018). Thus, our study aimed to assess the potential therapeutic use of Hamp against NNV infection in a highly susceptible species, the European sea bass. We selected the synthetic Hamp peptide derived from the European sea bass because: (i) it is induced at gene and protein levels upon NNV infection (Valero et al. 2020), (ii) exerts direct bactericidal and anti-NNV activity (Chia et al. 2010; Neves et al. 2015), (iii) regulates the immune response (Álvarez et al. 2016; Cervera et al. 2024) and (iv) reduces mortalities caused by NNV when administrated before viral challenge (Cervera et al. 2024).

Hamp treatment affected the course of the disease, increasing the survival rates and decreasing the number of fish suffering severe clinical signs. These results are in agreement to what occurs when administering Hamp or epinecidin to medaka or Nkl to European sea bass before NNV-infection (Valero et al. 2021; Wang et al. 2010a). However, in grouper infected with NNV, the therapeutic administration of Hamp and epinecidin failed to ameliorate survival rates (Wang et al. 2010b), suggesting differential effects of AMPs among species. Although the direct anti-NNV properties of AMPs have been probed for tilapia Hamp in vitro (Chia et al. 2010)*,* these effects were not observed in the in vivo studies. Our in vivo analysis showed no alterations in the transcriptional levels of *cp* or *rdrp* viral genes, suggesting normal viral transcription after Hamp treatment. In fact, NNV immunostaining in the brain revealed that the number of infected cells was not modified upon Hamp treatment even though the viral capsid protein levels were increased. Strikingly, injected synthetic peptides are quickly degraded at the injection site (Cervera et al. 2024). Therefore, we may eliminate the idea of a direct antiviral effect of Hamp against NNV in vivo, suggesting that the reduction of mortalities may be orchestrated through its immunomodulatory properties, as previously seen in our laboratory (Cervera et al. 2023, 2024).

We evaluated whether Hamp treatment was able to modulate the humoral immune response. AMPs, such as NKl, Dic and Hamp, are known to be induced upon NNV infection (Cervera et al. 2024). Moreover, the bactericidal activity is one of the easiest and most representative assays of the innate humoral immunity, which is carried out by several factors, including an array of AMPs (Shabir et al. 2018). For all these reasons, we analyzed this activity and AMP levels upon NNV infection. Unfortunately, the NNV-induced antibacterial activity was reduced by Hamp treatment in serum, brain and head kidney. However, in the target site of NNV replication, i.e., the brain, we observed increased levels of Nkl and Dic peptides and *nkl*, *hamp1*, *lyz* and *defb1* transcripts after Hamp administration, corroborating the cross talk among AMPs previously observed in European sea bass (Cervera et al. 2023, 2024). Considering that both Nkl and Dic peptides have been related with European sea bass antiviral responses (Valero et al. 2015, 2020), and the potential of Hamp to dismiss brain damage and preserve neurons (Vela 2018), it is reasonable to find higher survival rates and the prevention of the most severe clinical signs in our study.

It is well known that NNV infection elicits an exacerbated inflammation in the brain (Chaves-Pozo et al. 2019; Moreno et al. 2020), being the main cause for high mortalities. Hamp is known to have a role in the control of the inflammatory response in teleost fish (Katzenback 2015). In this study, Hamp-treated fish after NNV challenge greatly up-regulated anti-inflammatory cytokine *il10* in the brain and muscle and at the same time reduced pro-inflammatory cytokine *il6* in the muscle, similarly to what occurred during Hamp treatments in other fish trials (Álvarez et al. 2022; Cervera et al. 2023; Pan et al. 2011a). These data suggested that Hamp could diminish the exacerbated inflammatory response observed in the European sea bass brain upon NNV infection. In addition, Hamp treatment recovered the NNV-decreased *il6* and *mpo* levels in HK and increased the chemokine *il8* levels in spleen when compared with NNV-infected fish. These data are in agreement with the maintenance of some pro-inflammatory levels in immune tissues that allow cell recruitment to the infection site and also the antigen-presenting process in those tissues (Chaves-Pozo et al. 2005). Strikingly, Hamp reduced the muscle levels of genes encoding molecules related to leucocyte markers, activation and recruitment, but increased in the brain, leading to leucocyte recruitment at the NNV-replication tissue and favoring the local control of the inflammation, in particular, and the immune response, in general. Thus, CXCR3, expressed by many cell types including monocytes, dendritic cells, neutrophils, T or NK-like cells, endothelial cells, neurons and astrocytes (Valdés et al. 2022), was increased in the brain together with its interferon-gamma inducible ligand, *cxcl9*, and *il8*, *csf1r*, *cd4* and *ighm*. Thus, these data suggest the infiltration of leucocytes involved in the regulation of the inflammatory response, among others, in the brain. At the same time, Hamp administration reduced the expression of most of these genes in the muscle, suggesting that Hamp elicits a redistribution of leucocytes to drive the immune response to the brain. The different regulation of these molecules in the different tissues could orchestrate the effective response in the host that resulted in the survival levels observed in this study.

Conversely, AMPs are known to promote the adaptive response as well as link the innate and adaptive immunity (van der Does et al. 2019). Our data show that Hamp administration increased the transcription of *ighm* in the brain and reduced it in muscle, together with an increased production of total IgM in serum. These data suggest that B cells could be mobilized to the NNV-replication tissue, instead of the injection site, promoting the production and selection of specific antibodies. In fact, the transcription of leucocyte markers involved in antigen presentation and regulators of B cell (*cd4*) increased in the brain. Some studies have already demonstrated the role of fish AMPs as promoters of the adaptive response due to their adjuvant properties as described for pleurocidin (Liu et al. 2020), oreochromicin (Acosta et al. 2014), epinecidin-1 (Huang et al. 2011) or β-defensin (Zheng et al. 2023). Therefore, it is tempting to speculate that Hamp administration would show adjuvant properties and promising applications in teleost fish.

In conclusion, therapeutic administration of Hamp peptide upon European sea bass infection with NNV reduces fish mortality and the severity of the clinical signs. Our data showed that Hamp increases the presence of other AMPs with antiviral activity, such as Nkl and Dic, and induces an anti-inflammatory status that may control the exacerbated inflammatory response and prevent cell damage induced by NNV in the brain. In addition, Hamp seems to influence the redistribution of leucocytes to control brain inflammation and promote the antigen-presenting process and the generation of the specific response. Overall, the stimulation of the host immune response along with the increase in survival postulated Hamp treatment as an excellent tool to be used in European sea bass farming against NNV outbreaks.

## Supplementary Information

Below is the link to the electronic supplementary material.

Supplementary file1 (DOCX 24 KB)

## Data Availability

Data will be available on request**.**
