# Supplementary material for: Hepcidin peptide controls the inflammatory response induced by betanodavirus infection and improves European sea bass (Dicentrachus labrax) survival
Source: Mar Life Sci Technol. 2025 Jan 23;7(1):110–9. doi: 10.1007/s42995-024-00262-w (PMC11871212; doi:10.1007/s42995-024-00262-w)
Supplement: Supplementary file 1 — Supplementary file1 (DOCX 24 KB) [file 42995_2024_262_MOESM1_ESM.docx]

**Supplementary Table S1.** Primers used in this study.

|  | **Protein name** | **Gene name** | **Accession number** | **Sequence (5’**−**3’)** |
| --- | --- | --- | --- | --- |
| **House-keeping** | Elongation factor 1 alpha | *ef1a* | AJ866727 | F: CGTTGGCTTCAACATCAAGA  R: GAAGTTGTCTGCTCCCTTGG |
|  | Ribosomal protein S18 | *rps18* | AY831388 | F: TTCCTTTGATCGCTCTTAACG  R: TCTGATAAATGCACGCATCC |
|  | Ribosomal protein L13 alpha | *l13* | DT044539 | F:GCGAAGGCATCAACATCTCC  R:AGACGCACAATCTTGAGAGCAG |
| **AMPs** | Hepcidin 1 | *hamp1* | KJ890396 | F: AAGGCATTCAGCATTGCAGTTG  R: CCGCAACTGGAGTGTCATTG |
|  | Hepcidin 2 | *hamp2* | DQ131605 | F: CCAGTCACTGAGGTGCAAGA  R: GCTGTGACGCTTGTGTCTGT |
|  | Dicentracin | *dic* | AY303949 | F: GGCAAGTCCATCCACAAACT  R: ATATTGCTCCGCTTGCTGAT |
|  | NK-Lysin | *nkl* | KY801205 | F: GAAGAAACACCTCGGGGAAT  R: GCAGGTCCAACATCTCCTTC |
|  | Defensin beta 1 | *defb1* | DLAgn_00041270 | F: CCTTTCCTTGGTCTTGCCCA  R: ACACACAGCACAAGAAGCCT |
|  | Lysozyme | *lyz* | KJ433681.1 | F: ATTTCCTGGCTGGAACACAG  R: GAGCTCTGGCAACAACATCA |
| **Inflammation-related molecules** | Interleukin-10 | *il10* | DQ821114.1 | F: ACTCCTCGGTCTCTTCTCCT  R: TCCACAAAACGACAGCACTG |
|  | Interleukin-1 beta | *il1b* | AJ269472 | F: CAGGACTCCGGTTTGAACAT  R: GTCCATTCAAAAGGGGACAA |
|  | Interleukin-6 | *il6* | AM490062 | F: ACTTCCAAAACATGCCCTGA  R: CCGCTGGTCAGTCTAAGGAG |
| **Leucocyte-recruited molecules** | Interleukin-8 | *il8* | AM490063 | F: GTCTGAGAAGCCTGGGAGTG  R: GCAATGGGAGTTAGCAGGAA |
|  | C-X-C motif chemokine receptor 3 | *cxcr3* | ENSDLAT000050 | F: ATCCTGTACGCCTTTGTGGG  R: GTCGGCAGACTCAGACCAAA |
|  | CXC chemokine 9 | *cxcl9* | DLAgn_0001298 | F: TCTGTCAGCTCGCCTTTCTG  R: TTCGTACTTGGACACGCACA |
| **Leucocyte markers** | Myeloperoxidase | *mpo* | DLAgn_0011834 | F: GAAGAGTGGGGCCTTTGTTT  R: CTGGGCCTCAGTGAAGACTC |
|  | Macrophage colony-stimulation factor 1 receptor | *mcsf1r* | KM225787 | F: TTTCGGAAAGGTTGTTGAGG  R: TCTCATCTGAATGGGCACTG |
|  | T-cell receptor beta chain | *tcrb* | FN687461 | F: GACGGACGAAGCTGCCCA  R: TGGCAGCCTGTGTGATCTTCA |
|  | Cluster of differentiation 8 alpha | *cd8a* | AJ846849 | F: CTGTCCTCCGCTCATACTGG  R: TTGTAATGATGGGGGCATCT |
|  | Cluster of differentiation 4 | *cd4* | AM849812 | F: ATTCTTTGCTAAGCCAGGCG  R: CATTGTCTTGGTCTGGCGTC |
|  | Immunoglobulin M heavy chain | *ighm* | FN908858 | F: AGGACAGGACTGCTGCTGTT  R: CACCTGCTGTCTGCTGTTGT |
|  | Non-Specific Cytotoxic Cell Receptor Protein 1 | *nccrp1* | AY651258 | F: ACTTCCTGCACCGACTCAAG  R: TAGGAGCTGGTTTTGGTTGG |
| **Antiviral response** | Interferon-induced GTP-binding protein Mx | *mx* | AM228977 | F: GTATGAGGAGAAGGTGCGTCC  R: CTCTTCCCCGAGCTTTGGTC |
| **NNV** | NNV coat protein | *cp* | D38636 | F: CAACTGACAACGATCACACCTTC  R: CAATCGAACACTCCAGCGACA |
|  | Protein A | *rdpr* | AF319555 | F: GTGTCCGGAGAGGTTAAGGATG  R: CTTGAATTGATCAACGGTGAACA |
